# Ecology of fear: predator avoidance reduces seed dispersal in an ant

**DOI:** 10.1098/rsos.230530

**Published:** 2023-07-19

**Authors:** Dumas Gálvez

**Affiliations:** ^1^ Coiba Scientific Station, City of Knowledge, Calle Gustavo Lara, Boulevard 145B, Clayton 0843-01853, Panama; ^2^ Smithsonian Tropical Research Institute, Panamá PO Box 0843-03092, Balboa, Ancón, Panama; ^3^ Programa Centroamericano de Maestría en Entomología, Universidad de Panamá, Estafeta universitaria, Avenida Simón Bolívar, 0824 Panama City, Panama; ^4^ Sistema Nacional de Investigación, Panama City, Panama

**Keywords:** *Ectatomma ruidum*, fear, learning, *Rhinella alata*, myrmecochory, three-dimensional-printed model

## Abstract

The ecology of fear refers to the non-fatal cost that predators and parasites impose on prey populations. These non-consumptive effects (NCEs) can influence animal–plant interactions, but evidence thereof comes mainly from vertebrate systems with less focus on invertebrates. Here, I investigated whether the foraging behaviour of the ant *Ectatomma ruidum* was influenced by its primary predator, the forest toad *Rhinella alata*. In field tests, the probability of seed removal by the ants was 25% for seeds placed with the forest toad compared to 32% for control seeds, suggesting that toads reduce ant foraging rates. A further experiment revealed that ants which had previously encountered the predator and its faeces were more likely (59%) than inexperienced ants (50%) to avoid the exit with the predator faeces. This outcome suggests that ants are capable of learning cues associated with predation risk, possibly leading to NCEs. This indicates that predators can exert NCEs on invertebrate prey with potential cascading effects on seed dispersal, extending results previously seen only in vertebrate seed dispersal systems.

## 1. Introduction

The ecology of fear refers to the negative non-consumptive effects (NCEs) that predators impose on prey as a result of modification of their behaviour and physiology to avoid predation [1,2]. NCEs in arthropods are well established and include increased oxidative damage, decreased escape performance [3], reduced oviposition [4], reduced survival [5], reduced mass gain, increased development time and increases of the stress neurohormone octopamine [6] among others. Importantly, NCEs can even drive natural selection on prey populations by generating non-consumptive mortality [7].

The effects of NCEs on arthropod–plant interaction systems are relatively poorly documented [8], although some studies have observed that the presence of predators influences the rates of decomposition [9] and herbivory levels [10–12]. Seed dispersal by ants (myrmecochory) may also be influenced by NCEs; myrmecochory is ecologically important and at least 11 000 species of angiosperms worldwide depend on it for successful establishment and reproductive success [13]. Myrmecochorus plants make use of their seeds' lipid-rich appendages (elaiosome) and chemicals to induce ants to carry the seeds to their nest, where the elaisome is used to feed the larvae and the seeds are then discarded inside or outside the nest [14]. As demonstration of the potential importance of NCEs on myrmechochory, Tanaka *et al.* [15] documented an example where ants generated NCEs on seed predators, and increased the chances of seedling establishment.

Ants are often studied in the context of being predators [8] rather than prey and the literature contains no work on the effects of fear on the behaviour of ants as seed dispersers, contrasting with the robust body of evidence documented for vertebrate systems [16]. In this study, I investigated the impact of the native forest toad *Rhinella alata* on the seed dispersal behaviour of the ant *Ectatomma ruidum*, in natural and laboratory settings. At least in Panama, *E. ruidum* is the main prey of *R. alata* [17] and this ant is a seed disperser of several plant species in the neotropics [18–20], including non-myrmecochorous plants [18,21] thought to be important to the restoration of disturbed environments [22]. Therefore, understanding NCEs acting on *E. ruidum* can provide new insights into how plant–ant interactions and forest dynamics are influenced by ant predators.

To address this question, I first quantified seed dispersal rates by ants in the presence or absence of the toad in the field. Next, since presence of the toad reduced seed removal, I decided to evaluate the potential mechanism acting on the seed dispersal results. For that, I performed a Y-maze choice experiment in the laboratory comparing the effects of visual and non-visual cues by which the ants may have responded to the toad. This experiment intended to elucidate whether an innate response was involved in the response; for instance, expecting that vision would play a major role for the ants [23]. In this experiment, one of the maze's exits always contained an unobstructed exit (control) and the other exit contained a three-dimensional (3D)-printed toad in one of four possible combinations: a static toad, a moving toad, a static toad with faeces, or a moving toad with faeces. Finally, in a following experiment, I investigated whether ants could learn to associate predation risk with the predator's faeces—as an indication of NCEs—by performing the Y-maze experiment (control versus faeces) on ants that had or had not previously encountered the predator. Overall, the study of hard-wired and learned responses (experiments 2 and 3, respectively) in predator–prey models is rarely explored [24], and the *E. ruidum*–*R. alata* system may shed some light into predator–prey interactions in insects.

## 2. Material and methods

### 2.1. Experiment 1: seed dispersal

In this experiment I compared rates of seed removal from paired cages in the Pipeline Road area of Soberania National Park in Panama, near the Juan Grande creek, in which both ants and toads are common. I used *Psidium guajaba* seeds (common guava), which are not a myrmecochorous plant in the strict sense of possessing an elaiosome. However, *E. ruidum* is an important seed disperser of non-myrmecochorous plants [18,21,22], including seeds with fleshy pulp [18], similar to guava fruits.

**Figure 1.**
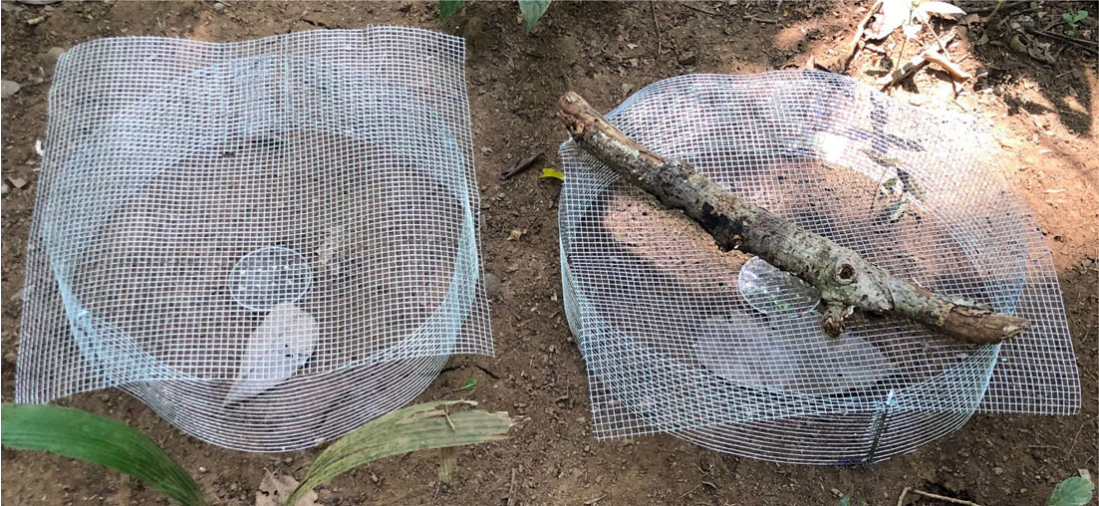
Field experiment setup to test the effect of the toad *Rhinella alata* on seed dispersal by the ant *Ectatomma ruidum*. Left and right cages show the control and toad treatment, respectively.

Each cage pair consisted of two treatments: a treatment cage containing an adult toad and a paired control cage without a toad; the two cages were placed 10 cm away from each other and I performed 34 replicates (pairs of cages) with unique toads. All cages had entrances of sufficient size for the ants to enter (diameter: 50 cm, height: 11 cm, mesh hole: 0.6 cm × 0.6 cm; figure 1). All cages were also furnished with a dead leaf under which a toad could potentially hide. In each replicate, I located both cages at approximately the same distance from the nearest ant nest. No single nest had access to more than one replicate, as these were separated by five to ten metres. Within each cage 10 seeds were haphazardly placed in grids of 2 × 5 on Petri dish lids with a diameter of 90 mm. Seeds were isolated manually from a single fruit and each seed was left with remnants of the fruit pulp. Seed removal was monitored every five minutes for two hours with two observers assisting the monitoring. At the end of the experiments, I confirmed that no seeds remained around the Petri dish lid. I performed this experiment on four different dates (8–9 unique replicates per date: 29 January, 1–3 February 2021) and the toads were immediately released at the end of each experiment. On the last three dates, I estimated ant visit rates by counting the number of ants at each time interval.

I carried out a Cox proportional hazard analysis (*coxph*) in the survival package in R [25,26] to compare the survival time of seeds placed with a toad or in the control condition by specifying a paired analysis of cages with the function *frailty*. I used a generalized linear mixed-effects model with a Poisson distribution to test whether ant visit counts differed between control cages and cages with toads, specifying pair of cages (ID) as a random effect [27]. I did not find evidence of overdispersion, as assessed using the function *dispersion_glmer* in the blmeco package.

### 2.2. Laboratory experiments

#### 2.2.1. Experiment 2: mechanisms (visual versus non-visual stimuli)

**Figure 2.**
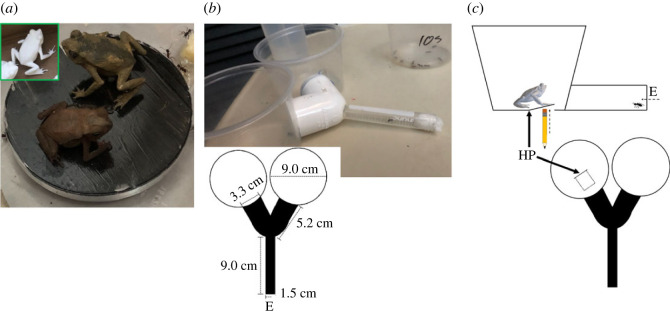
Illustration of 3D-printed models of the toad *Rhinella alata* and Y-maze. (*a*) Unpainted model (green square) and painted model (top) next to a live toad (bottom), for comparison, in a box containing *E. ruidum*. (*b*) Photograph of Y-maze and top view of the Y-maze diagram in which ants were exposed to two exits at a time. White circles represent plastic containers. One exit always remained empty (control) and the other one contained a 3D-printed toad presented in one out of four possible combinations (static toad + faeces, static toad − faeces, moving toad + faeces or moving toad − faeces). (*c*) Details on the moving toad treatment: top and side views of one exit in the Y-maze containing a 3D-printed model *of R. alata*. The simulation of a moving toad was done by pushing this semi-lid manually with the pencil up at a frequency of one cycle per second. The semi-lid lowers by itself with the weight of the toad. HP shows the hinge point and E the entrance of the Y-maze. The container containing the treatment was placed on the edge of a table and the moving motion of the hand was done from underneath the table; therefore, it is unlikely that the ants could see the hand.

Ten ant colonies were collected in the forest of Paraiso, Panama (9.0327° N, 79.6263° W), a secondary forest which is surrounded by the Camino de Cruces National Park. The colonies contained between 50 and 100 workers. Importantly, in more than two years of personal observation (2019–2021), I never observed *R. alata* in the Paraiso forest, suggesting that the ants employed for this experiment did not have any previous exposure to this predator. Colonies were kept in plastic boxes (11.5 × 18.5 × 31.5 cm^3^) for around a week before the experiments, and provided with ad libitum water and food made of honey, eggs and agar. The room was under a 12 : 12 white light cycle at 27°C and *ca* 55% humidity. I carried out the experiments on 6 April 2021 and I used identical 3D-printed models of *R. ralata* that were coloured with acrylic paint (figure 2*a*), following approaches from other studies that used 3D-printed toads to study behavioural responses [28]. A Y-maze (figure 2*b*) selection experiment was used to examine whether visual or non-visual cues drove the seed dispersal results. Y-mazes have been used extensively to study ants' behaviour and are regarded as a valid method to study memory and cognition in animals [29,30]. Ants had to try to leave the maze through one of two arms in the maze. One exit always remained unobstructed, as a control, and the other one contained a 3D-printed toad presented in one of four possible combinations: (1) static toad with faeces, (2) static toad without faeces, (3) moving toad with faeces, and (4) moving toad without faeces (see figure 2*c* for details on the moving toad). If non-visual stimuli (olfactory or chemotactile: contact dependent) influence ant foraging behaviour, I expected the cue to produce less selection of that exit, regardless of whether the toad was stationary or moving. However, if visual stimuli were more significant, then ants would exit the maze less frequently through the exit with the presence of a moving toad. This is independent of the presence of faeces. Another possibility was that ants could distinguish the toads by sight alone, without olfactory, chemotactile or movement stimuli, as evidenced by lower selection of any toad combination as compared to the control exits. Each ant was introduced into the tunnel through the entrance (E in figure 2*b*,*c*) and the test started as soon as the ant entered. To remove bias due to exit position or olfactory–chemotactile traces left by previous ants, I switched the position of the exits and cleaned them with 70% ethanol after two ants completed a test and at the end of all replicates. I performed ten replicates of all toad combinations and used ten ants per replicate (100 ants per treatment), by using one ant at a time. The ten ants from a replicate came from the same colony. *Ectatomma ruidum* can use both individual and group foraging [31]. Faeces were collected 24 h before the experiments with use of forceps from ten toads that were kept individually in plastic boxes and were fed daily with workers. Each toad provided faeces for the two treatments with faeces and I used pellets from approximately the same size in each replicate. Treatment order was randomly assigned, and each ant was used only once. I terminated each test after one minute, in the event that an ant did not leave the tube.

I used generalized estimating equations (GEE, function *geeglm*), in the *geepack* package in R, with binomial distribution to analyse the relationship between selection of an exit (1: empty, 0: toad), toad treatment (static toad − faeces, static toad + faeces, moving toad − faeces and moving toad + faeces) and arm side (fixed effects). I specified the colony of origin as the cluster to account for the lack of independence of ants per replicate and non-responsive ants were excluded from the analysis [32]. I used a binomial test to compare the frequency of choosing the empty exit versus the exit with a toad (pooled data of all toad treatments).

### 2.3. Experiment 3: learning

To test whether the naive ants could learn to associate a predator's olfactory cues with predation risk, I placed a caged toad with ad libitum water in the ants' box for 48 h, together with samples of faeces from the same toad. These were termed ‘experienced’ ants as compared to control ants that were not in contact with the toad, referred to here as ‘inexperienced’ ants. The mesh size allowed the ants to enter the cage and the toad was able to feed on those ants, which also resulted in new faeces. After the 48 h, I ran the experiments with the Y-maze to compare the control versus faeces exit usage in both experienced and inexperienced ants, then replicated the experiment 10 times (10 ants per replicate: 100 ants), by running one ant individually in the maze; all ants from a replicate came from the same colony. Each colony provided ants for both treatments and experienced ants from each colony were exposed to a unique toad. I switched the position of the exits and cleaned with 70% ethanol as described before. I used GEE with binomial distribution as described before to analyse the relationship between selection of an exit (1: empty, 0: faeces), exposure treatment (experienced versus inexperienced), arm side and the order in which the replicates were run (fixed effects). I specified the colony of origin as the random effect and non-responsive ants were excluded from the analysis.

## 3. Results

### 3.1. Experiment 1: seed dispersal

**Figure 3.**
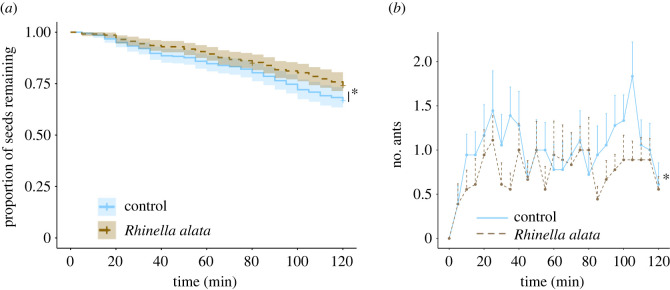
Field experiments. (*a*) Survival curves of seeds that were placed with a toad in the cage or in the control cages in the field (*n* = 34). Shading represents 95% confidence intervals. (*b*) Number of ants counted every five minutes for two hours in each cage type (*n* = 26). Bars represent standard errors. *Significant differences.

Seeds placed with a toad were removed in a lower proportion than seeds in the control cages (25 and 32%, respectively; *X*^2^ = 15.8, d.f. = 1, *p* < 0.0001; figure 3*a*). Visit rate increased in both treatment groups during the first 30 min and ants displayed lower visit counts to the cages with the toad than to the control cages (z = −3.9, *p* < 0.0001, *n* = 27; figure 3*b*).

### 3.2. Experiment 2: mechanisms

**Figure 4.**
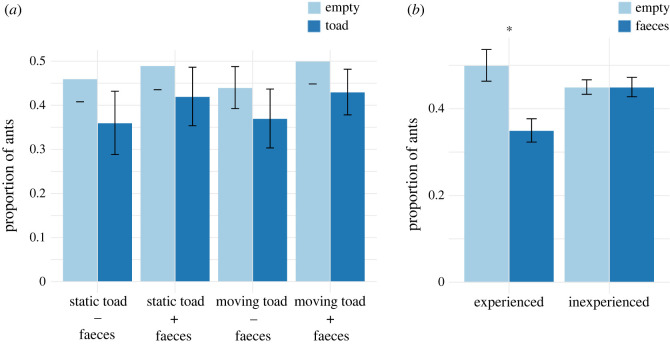
Y-maze experiments. (*a*) Proportions of ants walking through an empty exit versus an exit containing one of four experimental treatments: a static toad (static − faeces), a moving toad (moving − faeces), a static toad with faeces (static + faeces), or a moving toad with faeces (moving + faeces). (*b*) Proportion of experienced and inexperienced ants that walked through an exit with the predator's faeces or the empty exit. *Significant differences. Bars represent standard errors.

For the inexperienced ants used in this portion of the study, the probability of choosing the empty exit versus the exit containing the model toad did not differ significantly among the four experimental treatments (Wald = 0.17, *p* = 0.98; figure 4*a*; electronic supplementary material, table S1) or between when the model toad was placed in the left arm versus the right arm of the Y-maze (Wald = 0.24, *p* = 0.63). Overall, ants were not more likely to choose the empty exit versus the exit with the toad (binomial test, *n* = 347, *p* = 0.2).

### 3.3. Experiment 3: learning

The proportion of ants walking through the predator faeces exit versus the empty exit was lower for experienced ants than for inexperienced ants (Wald = 5.8, *p* = 0.02; figure 4*e*), but did not differ according to whether the faeces were in the left arm versus the right arm of the Y-maze (Wald = 0.01, *p* = 0.97) or in relation to replicate order (Wald = 1.44, *p* = 0.23; electronic supplementary material, table S2).

## 4. Discussion

I am not aware of any study on the effects of NCEs on seed removal or dispersal by an insect. However, in this study, ants reduced foraging activity in the field when the toad was present, consequently resulting in lower seed removal rates, analogous to the interactions between prey and predator in vertebrate systems [33]. Despite this outcome, the mechanism by which toads induce lower foraging in the ants remains unclear, which is a surprising result because the ant often responds to movement from an observer in the field by hiding or remaining static. It is however clear that after a relatively short exposure period to the predator and its faeces in the laboratory, ants learned to associate predation risk with the predator's faeces, suggesting that NCEs can in fact occur. Furthermore, the reduced rate of ant visits to the cages with the toads and the fact that no predation events were witnessed by three observers also suggest that NCEs can take place in the field. Moreover, *R. alata* as a sit-and-pursue predator [34] remains sedentary during long periods which should generate strong cues resulting in NCEs for the ants [35].

The experiments employed to clarify the NCEs' mechanisms were done in the laboratory, suggesting that some factor(s) missing in the laboratory influence experimental detection, the 3D models do not generate a realistic signal or previous exposure to the predator is required, as seen in the learning experiment. This also suggests that the avoidance behaviour is not an innate response, as seen in naive tephritid fruit flies exposed to volatile cues of spiders and one ant species [36]. Evidence of ants learning to avoid predators comes from ant–antlions systems [37,38] and such trait of the ants could be adaptive for encountering novel predators in new environments and perhaps it contributes to the explanation of why this ant species is abundant in the neotropics [39]. Besides, rapid learning in *E. ruidum* occurs under multimodal stimuli during foraging [36] and it might occur also in the context of predation risk, which merits further work.

The perception of risk influences myrmecochory rates and if rapid seed removal is imperative to reduce seed predation [40], then reduced foraging in areas of predation risk for ants could affect the plant's reproductive success. For instance, seed manipulation by *E. ruidum* increases germination success and reduces the time to germination [19]; therefore, lack of access or delayed access may have negative effects on seedling establishment for some plant species. Moreover, this toad–ant interaction could generate other cascading effects, for example acting on brown food webs [41] by lowering predation by ants with increases in toad density. In a similar vein, when toads are abundant, nontarget or less-preferred ants [17] may benefit by obtaining access to resources that would be normally dominated by *E. ruidum*, like the effect caused by large carnivores favouring certain invertebrates on shorelines [42]. In one instance, several smaller ants (possibly *Trachymyrmex*) were observed dispersing seeds from one of the cages with toads (removed replicate). Further work is needed to evaluate these potential cascading effects.

It is also expected that predation risk for ants will change with seasonal fluctuations and across sites with varying levels of toad abundance [43]. This adds a spatio-temporal element to the distribution of risk, as seen in vertebrates [44]. Additionally, *R. allata* is a diurnal species [45], and thus colonies showing temporal plasticity in foraging [46] may benefit from night foraging. In vertebrate systems, prey species can be more active during periods of lower predators' activity [47] and shift their activity patterns in response to predators’ presence [48]. *Ectatomma ruidum* can also forage above the ground [49] and colonies with more arboreal foraging—when toads are abundant—would be under lower predator pressure, in a way analogous to the evolution of arboreality in vertebrates as an escape from predators [50]. Any potential shift or reduction in ants' foraging could negatively influence a myrmecochorus plant's fitness since they often show synchrony in fruit dehiscence with the period of maximum foraging activity [51]. Again, future work should evaluate whether foraging activities in *E. ruidum* vary or adapt spatially and temporally in response to densities of *R. allata*, to reduce overlap in activity with the predator.

Overall, this study suggests that the ecology of fear acting on seed dispersers may be widespread among vertebrate and invertebrate prey species. However, theoretical and experimental work comes mostly from vertebrate systems [16,52,53] which highlights the need for more work on ants and other invertebrates. A crucial element of myrmecochory is the sociality element of ants and future theoretical models should account for that and its interaction with the ecology of fear. As a result of the learning experiment, some questions arise, such as whether all the ants experienced the predator or if the information was propagated within the group [54] in the field and laboratory.

## Data Availability

All data supporting this paper are available as electronic supplementary material [55].
